# Memristive In‐Memory Object Detection with 128 Mb C‐Doped Ge_2_Sb_2_Te_5_ PCM Chip

**DOI:** 10.1002/advs.202505678

**Published:** 2025-07-17

**Authors:** Chenchen Xie, Yuqi Li, Longhao Yan, Sannian Song, Houpeng Chen, Ruijuan Qi, Xi Li, Yihang Zhu, Lianfeng Yu, Bonan Yan, Yaoyu Tao, Gaoming Feng, Yuchao Yang, Zhitang Song

**Affiliations:** ^1^ State Key Laboratory of Functional Materials for Informatics Shanghai Institute of Microsystem and Information Technology Chinese Academy of Sciences Shanghai 200050 China; ^2^ Beijing Advanced Innovation Center for Integrated Circuits School of Integrated Circuits Peking University Beijing 100871 China; ^3^ Center for Brain Inspired Chips, Institute for Artificial Intelligence Peking University Beijing 100871 China; ^4^ Shanghai Integrated Circuit R&D Center Shanghai 201210 China; ^5^ Guangdong Provincial Key Laboratory of In‐Memory Computing Chips School of Electronic and Computer Engineering Peking University Shenzhen 518055 China; ^6^ Center for Brain Inspired Intelligence Chinese Institute for Brain Research (CIBR) Beijing 102206 China

**Keywords:** in‐memory computing, mixed precision, object detection, phase change memory

## Abstract

Object detection, as a fundamental task in computer vision, mainly performs the classification and localization of objects in images or videos. However, traditional edge computing platforms fall short of meeting the demands for state‐of‐the‐art object detection model size and computing power. Here, a 128 Mb phase change memory chip is fabricated with a high memory yield of 99.99999% in a 40 nm node and utilized for efficient in‐memory vector‐matrix multiplication and in‐memory max computation. In particular, in order to mitigate the significant programming energy overheads for large‐scale memristor arrays and the reliance on high‐precision analog‐to‐digital‐converter (ADC) in compute‐in‐memory operations, a novel mixed‐precision weight mapping strategy is adopted. Compared with traditional schemes, the ADC modules achieve up to a 22.3× reduction in energy consumption while maintaining equivalent network performance. Ultimately, this memristive in‐memory object detection system demonstrates 4,180× higher energy efficiency and 228× greater computational throughput compared to GPU implementations.

## Graphical abstract

A memristive in‐memory object detection system is presented for edge computing based on a 128 Mb phase change memory chip (40 nm, 99.99999 % yield) enabling in‐memory vector‐matrix multiplication and max computation. A novel mixed‐precision weight mapping reduces analog‐to‐digital‐converter energy by 22.3×. This memristive in‐memory object detection system achieves 4,180× higher energy efficiency and 228× greater throughput than GPU implementations.

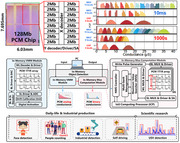

## 1 Introduction

As a fundamental problem in computer vision, object detection (OD) is the basis of complex computer vision tasks and has been widely applied in daily life, industrial production, scientific research, and other scenarios. Lately, deep learning has gained widespread popularity in the field of OD, primarily due to the availability of powerful computational devices, notably GPU.^[^ ^1^ ^]^ Hence, a multitude of promising methodologies have emerged for OD utilizing deep learning, including single‐shot multi‐box detection,^[^ ^2^ ^]^ Region‐based Convolutional Neural Networks (R‐CNN),^[^ ^3^ ^]^ Faster R‐CNN,^[^ ^4^ ^]^ and you‐only‐look‐once (YOLO)^[^ ^5^ ^]^ etc. Different from classification tasks, OD is highly demanding in model size and precision and has a model size of normally 100 Mb for high accuracy. Classical OD algorithms typically consist of two fundamental steps: the prediction step and the post‐processing step. CNN has strong feature extraction and generalization capability and is thus widely used as the backbone feature extraction network in OD algorithms. Besides, OD tasks require a non‐maximum suppression (NMS) algorithm for the selection of the optimal frame in order to complete the final object localization.^[^ ^6^, ^7^ ^]^

Both CNN in the prediction step and NMS in the post‐processing step require huge computing and storage resources. However, conventional computing architectures consist of issues such as von Neumann bottleneck and difficulties in data access which impose limitations in the deployment of OD algorithms on edge devices. Therefore, an energy‐efficient, large‐capacity, and high‐throughput computing platform is desired to meet the rapidly increasing demands for OD on the edge.

In order to address the challenges posed by conventional computing architectures, advanced computational architectures, such as in‐memory computing^[^ ^8^, ^9^, ^10^, ^11^, ^12^ ^]^ and neuromorphic computing^[^ ^13^, ^14^, ^15^, ^16^ ^]^ are currently under intensive research. Memristor, as an emerging electronic device, has been regarded as having significant potential in the development of advanced computing architectures. By utilizing the non‐volatile memory physical characteristics and multi‐level storage capability of memristive devices for both storing and computing, extensive information processing operations can be performed near or even in memory, effectively reducing the energy consumption associated with data transmission between memory and processors in von Neumann architectures.^[^ ^17^, ^18^, ^19^ ^]^ Recently, a large number of chips via the use of compact crossbar arrays of non‐volatile memristive devices with variable conductance states, including resistive random access memory,^[^ ^20^, ^21^, ^22^, ^23^ ^]^ phase change memory (PCM)^[^ ^24^, ^25^, ^26^ ^]^ and magnetic random access memory,^[^ ^27^ ^]^ etc., have been manufactured for establishing an energy‐efficient and high‐throughput computing platform for executing CNN inference, where can be hardware acceleration of vector‐matrix multiplication (VMM) by Ohm's law and Kirchhoff's current law. As a fundamental operator, ranking is executed numerous times daily in contemporary computing systems. Consequently, extensive attention has been dedicated to research endeavors associated with the development of efficient software and hardware co‐design tailored for ranking. In‐memory ranking,^[^ ^28^, ^29^, ^30^ ^]^ utilizing the binary conductance characteristics of the devices, accomplishes the ranking operation by combining the crossbar arrays with peripheral circuits, which leads to a substantial increase in ranking speed and a significant reduction in energy consumption. Based on in‐memory ranking methods, techniques for efficient in‐memory maximum value search can also be developed.

In this work, we introduce the concept of a memristive in‐memory OD system as a solution to pertinent issues. This is motivated by the discovery that a memristive crossbar array can be employed to efficiently perform two fundamental in‐memory computing operators in OD algorithms: in‐memory vector VMM and in‐memory max computation, corresponding to CNN and NMS operations, respectively. The expectation is that by following this advanced computing architecture, we can attain a significant level of processing speed and energy efficiency while retaining the capability to achieve arbitrarily high computational accuracy. We demonstrate the implementation of our memristive in‐memory OD system utilizing the YOLOv2 algorithm, based on a 128Mb carbon‐doped Ge2Sb2Te5 (C‐GST) PCM chip fabricated using a 40 nm foundry process with a high memory yield of 99.99999%, providing evidence for the feasibility of this concept. Memory yield is a critical performance metric for memory devices, with specialized test equipment, procedures, and standards established to quantify this parameter. Both traditional computing architectures and in‐memory computing impose stringent requirements on memory yield. By rigorously measuring memory yield, we ensure that the vast majority of cells on each wafer meet their performance specifications. This, in turn, gives us confidence that when object detection algorithms are deployed on large‐scale PCM arrays, they will satisfy the strict accuracy demands of in‐memory computing and achieve high computation yield in real‐world systems. To mitigate the huge parameter mapping overheads, we have developed a mixed‐precision parameter mapping, device programming, and analog‐to‐digital‐converter (ADC) configuration strategy so as to support OD models with a huge number of weights. Compared with GPU platforms. Our memristive in‐memory OD system improves the energy efficiency and throughput by 4180 times and throughput by 228 times.

## 2 Result and Discussion

### 2.1 The Concept of Memristive In‐Memory OD System

**Figure 1:**
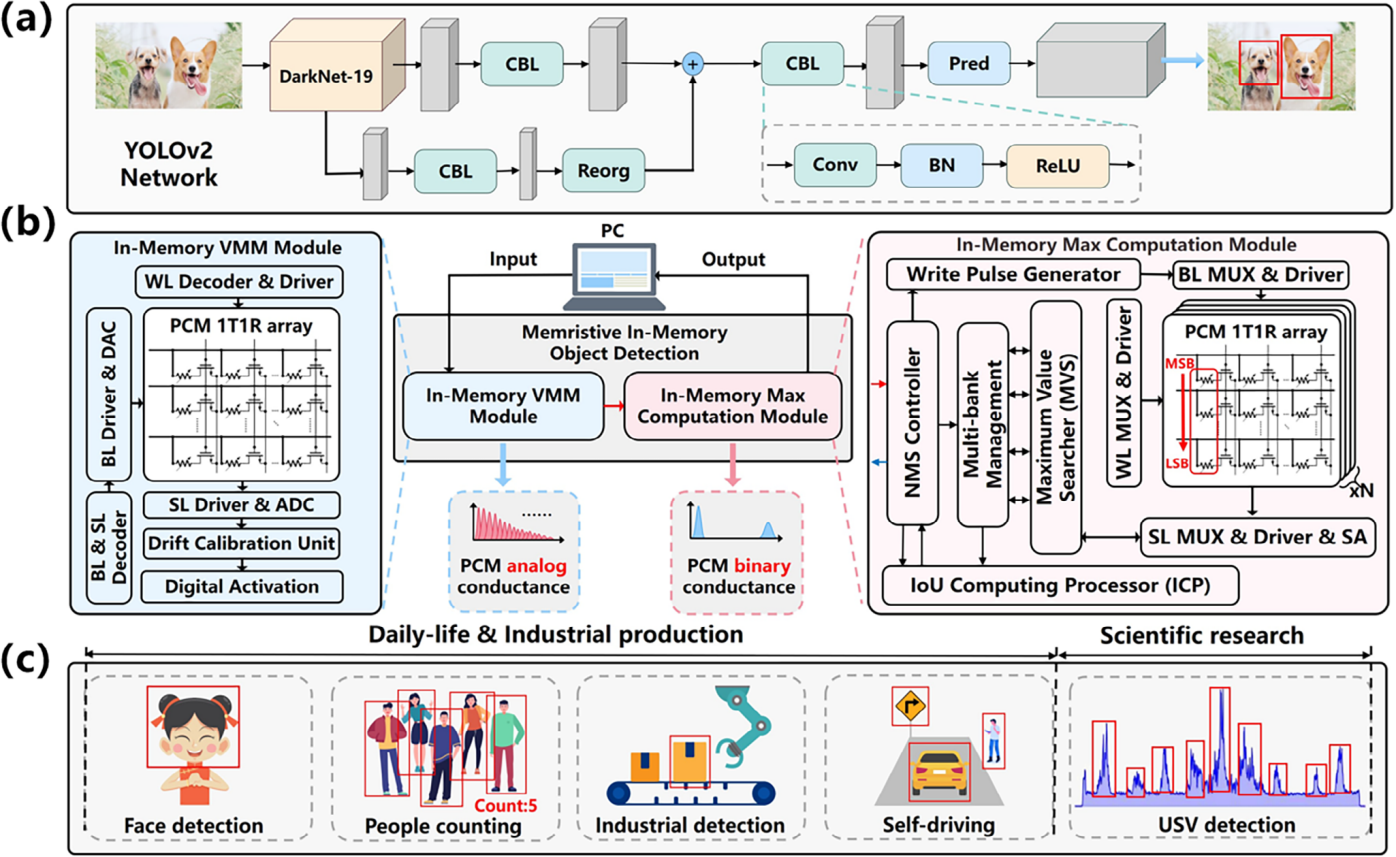
Introduction to PCM‐based memristive in‐memory OD system and applications. a) Network architecture of famous OD algorithm (Yolov2). b) Architecture of PCM‐based memristive in‐memory OD system and characteristics of PCM used in this system. c) Daily‐life & industrial production and scientific research applications of PCM‐based OD. In this work, we take advantage of the characteristics of PCM to implement an in‐memory VMM module and an in‐memory max computation module. Finally, the two are combined and applied to the OD at the same time, leading to significant improvement in energy efficiency.

OD algorithms, as illustrated utilizing the YOLOv2 algorithm in the case,^[^ ^31^ ^]^ can be divided into two major steps, the prediction step and the post‐processing step. The prediction step is mainly based on CNN, which extracts image features and classifies the object identified in the box. As shown in **Figure** **1**a, the DarkNet‐19 backbone consists of six different DarkNet blocks with several 3 × 3 convolutional kernels each and 2 × 2 maxpool kernels partly, is capable of classifying 32 × 32‐pixel RGB images into one of ten classes on the CIFAR‐10 dataset. The DarkNet‐19 backbone can be mapped as the conductance of PCM devices. In the former step, the input image is processed by CNN based on the DarkNet‐19 backbone, forming a tensor containing the significant information for the following process. Subsequently, employing diverse activation functions on the output tensor facilitates the extraction of positional information, categorical information, and confidence scores, which can further be utilized to generate prediction boxes and determine object category information based on the confidence scores.

The post‐processing step is based on the NMS algorithm, which selects the optimal box out of candidate boxes identified by CNN. The NMS algorithm first iterates through all candidate boxes, setting the confidence score of each candidate box to 0 if it falls below a predetermined threshold. Subsequently, the algorithm ranks the candidate boxes based on their confidence scores and identifies the candidate box with the highest confidence score, denoted as Box_max_, which can be performed within the memristive crossbar arrays. Following this crucial operation, the algorithm computes the Intersection Over Union (IoU), which represents the degree of overlap between two boxes, for all remaining candidate boxes (Box_i_) in comparison to Box_max_. When the IoU exceeds a specified threshold, it indicates a high degree of overlap between the candidate box (Box_i_) and Box_max_, prompting the removal of the candidate box (Box_i_). In the denouement of the post‐processing step, the algorithm determines object category information contained in each candidate box and outputs the detection results with a confidence score >0. The two steps are combined to complete the classification and localization of objects.

The primary principle of the memristive in‐memory OD system involves storing the parameters such as weights of the CNN in the prediction step and confidence scores of the NMS algorithm in the post‐processing step as the conductance values of memristive devices, which can encode values within an analog range or can be binary on crossbar arrays. The OD operation is accomplished through the combined use of the memristive crossbar arrays and peripheral circuits. In this work, we chiefly utilize PCM devices, which can be programmed to achieve the desired conductance values by altering the amorphous (high‐resistance)/crystalline (low‐resistance) configuration within the devices. Furthermore, the conductance values, when the devices are in either the amorphous or crystalline phases, are capable of facilitating stable binary data storage. As established in previous research, the non‐volatile and dynamical characteristics of PCM devices have been demonstrated to be successfully employed in novel computing paradigms such as neuromorphic computing and in‐memory computing, which further emphasizes their substantial potential in the realization of memristive in‐memory OD systems.

Although high‐precision parameters, including weights and confidence scores, are always desirable for both CNN and NMS, they are typically mapped to PCM analog conductance states in memristive in‐memory OD system. Achieving precise representation of high‐precision parameters using conductance states often requires iterative closed‐loop “write & verify” (W&V) programming,^[^ ^32^, ^33^ ^]^ and the programming time and energy overheads can be extremely high and in fact impractical for large neural network models especially at the scale of >100 Mb. To address it, we have adopted an efficient mixed‐precision parameter mapping strategy, where the high‐precision state of PCM with more time and energy‐consuming W&V is used for in‐memory VMM computation where high weight precision is crucial, while the low‐precision state of PCM, or even a binary one, with time and energy efficient one‐shot programming (OSP),^[^ ^34^ ^]^ which is employed for efficient writing of PCM cells into four distinct conductance states, is utilized for in‐memory max computation where low parameter precision suffices, respectively, so as to mitigate the programming time/energy overheads while warranting the network performance simultaneously. The novel device programming and parameter mapping scheme allows for tuning the parameter precision of each step according to its parameter size and precision sensitivity of each step, which also reduces the requirement for the number of high‐precision ADC and further enhances the area and energy efficiency of the memristive in‐memory OD system. In the remaining part of this article, we will expound on the detailed designs of the memristive in‐memory OD system and present a complete concept of our memristive in‐memory OD system. Therefore, we propose leveraging the physical characteristics of PCM to accelerate two critical stages of OD algorithms, namely the prediction stage based on CNN and the post‐processing stage based on NMS algorithms, for constructing the memristive in‐memory OD system.

### 2.2 Device Characteristics of PCM‐Based OD System

**Figure 2:**
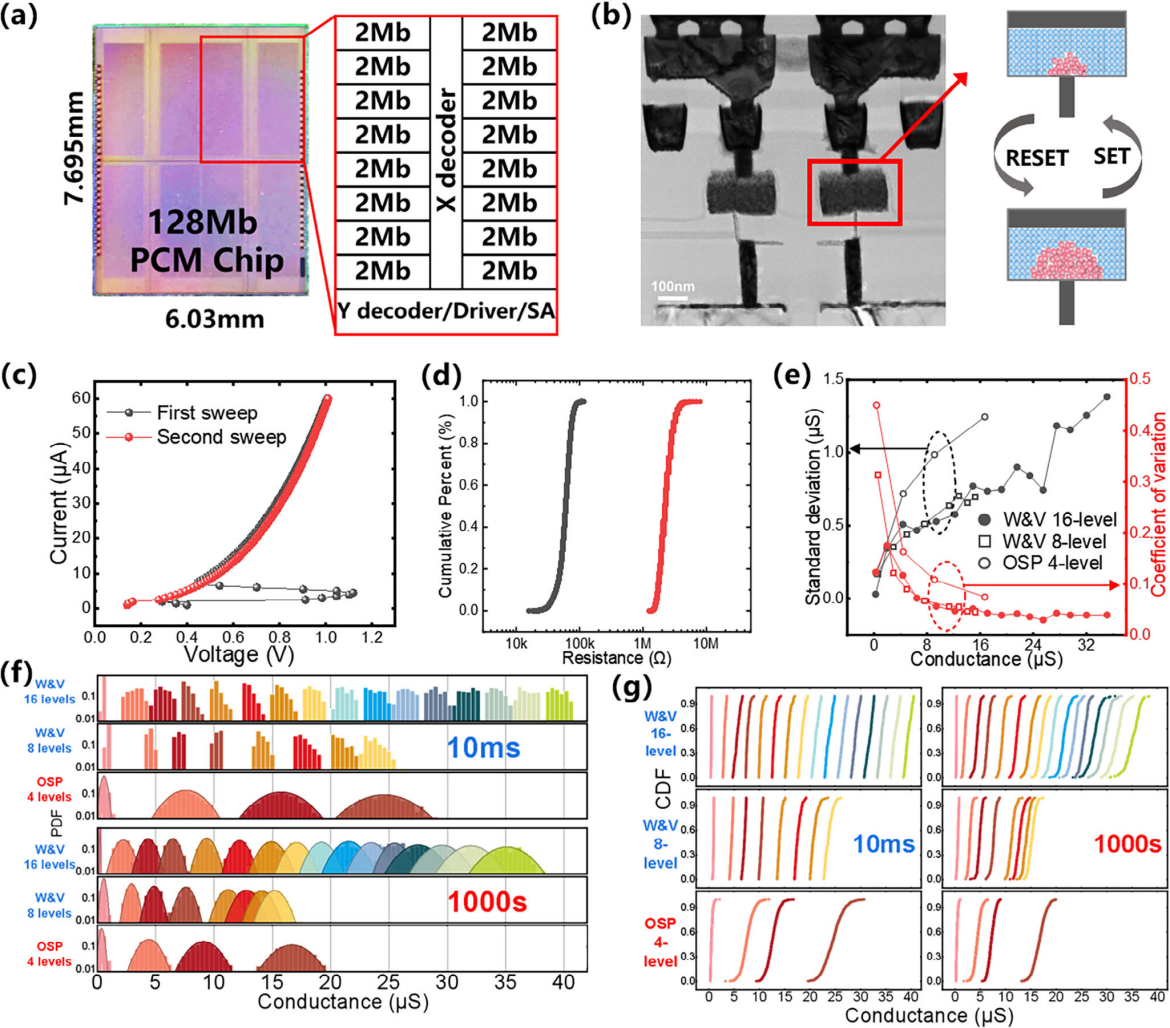
Device characteristics of PCM chip. a) The architecture of a 128 Mb PCM chip, consisting of four 32 Mb blocks. b) The TEM image of the 1T1R C‐GST PCM chip. c) *I–V* characteristics curve of PCM devices, the first sweep (black) is from 0 to 60 µA and the second sweep (red) is from 60 µA to 0. d) Digital characteristics of PCM, no overlap between two states. e) Standard deviation and coefficient of variation for 16, 8, and 4‐level states. f) Probability density function (PDF) plot of 4 states using one‐shot programming for analog conductance, 8 states, and 16 states with iterative programming to obtain more accurate conductance at 10 ms and 1000 s. g) Cumulative distribution function (CDF) plot of 4 states using one‐shot programming for analog conductance, 8 states, and 16 states with iterative programming to obtain more accurate conductance at 10 ms and 1000 s. One‐shot programming means that only one set operation or reset operation is used for programming (no iterative programming is used). The operating conditions are obtained through the summary of multiple tests in the previous stage. Iterative programming means that the conductance is programmed multiple times through the write‐verify‐rewrite method until it enters the target conductance range, which can obtain more accurate conductance than the OSP method.

For the proof‐of‐concept demonstration in the experimental showcase of the memristive in‐memory OD system, we utilized a 128Mb C‐GST PCM chip, intricately fabricated based on a 40 nm foundry process and comprising four 32Mb blocks, as depicted in **Figure** **2**a,b. Each block is in turn divided into sixteen 2Mb 1T1R arrays with peripheral circuits, including a decoder, driver, and sense amplifier. Figure 2c,d shows that the fabricated PCM chip achieves an on/off ratio of >10 and high uniformity by optimizing the C‐GST composition as well as the height and width of the blade‐type heating electrode. Furthermore, we carried out comprehensive tests on the full wafer (Figure S3, Supporting Information), and the overall device memory yield reached 99.99999%. To investigate the conductance precision of the device with different programming pulses, we explored the Set and Reset operations with different current pulse widths and amplitudes (Figure S4, Supporting Information). The primary method involved initially resetting the PCM device to a low conductance state, followed by the application of current pulses with different amplitudes to observe the conductance changes. We increased the width of the current pulses and repeated the aforementioned process, subsequently compiling the results. The results show that our PCM chip has abundant conductance states in the range of 0–40µS, and notably, the conductance window of the device broadens as the width of the current pulse is incrementally increased. This phenomenon is primarily ascribed to the set pulses raising the temperature of the phase‐change material to an intermediate range between its crystallization and melting thresholds, wherein extended pulse durations contribute to a more comprehensive crystallization process within the material. Figure 2f,g exhibits the conductance distributions at 10 ms and 1000s after the PCM devices are programmed to 16 levels (upper), 8 levels (middle) using W&V, and 4 levels using OSP (lower). We can conclude that the distribution of each state after conductance drift with the W&V scheme is more concentrated, while the states with the OSP scheme show wide distributions in both freshly programmed cells and after conductance drift. This is also indicated by Figure 2e showing the standard deviation (left y‐axis) and coefficient of variation (defined as standard deviation divided by mean, right y‐axis) of PCM cells versus the mean conductance of each state. The standard deviation, σ, enables a nuanced comparison of dispersion in conductivity distributions across corresponding conductive states, while the coefficient of variation simultaneously elucidates the uniformity of these distributions across different states.

### 2.3 In‐Memory VMM Module

**Figure 3:**
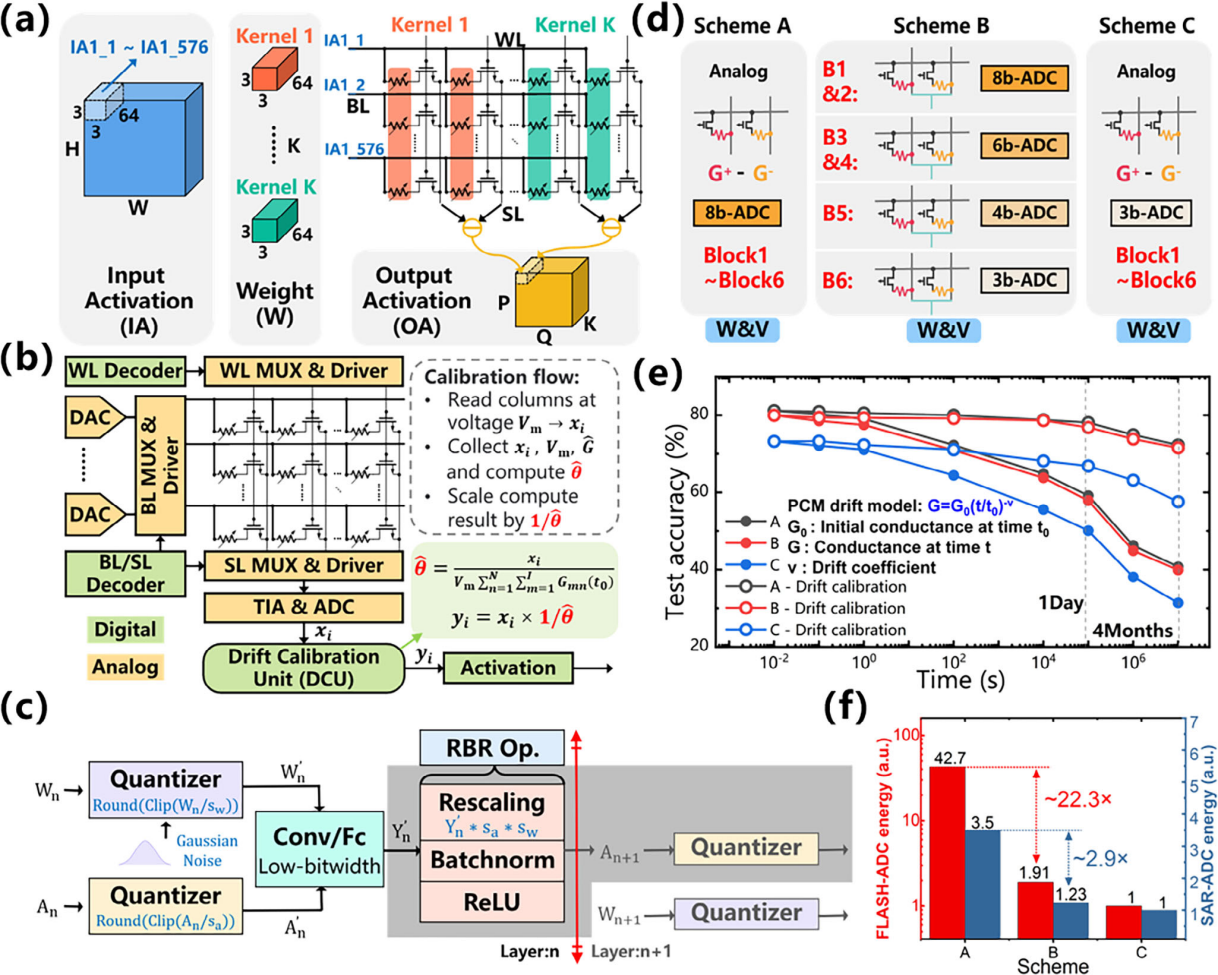
Architecture and performance of in‐memory VMM module. a) Array mapping method of CNN, convolution kernels are mapped to different columns, and differential conductance is used to represent a network weight. b) PCM‐based in‐memory VMM module architecture, analog VMM is completed in the array, drift calibration unit can achieve drift compensation periodically. c) Principles of model hardware‐aware training. d) Conductance mapping and ADC configuration schemes for different blocks in the DarkNet‐19 backbone. e) The test accuracy of conductance drift and drift calibration, the results of schemes A and B are very similar. f) Comparison of FLASH‐ADC and SAR‐ADC energy consumption of 3 schemes.

The convolutional kernel weights of all layers in DarkNet‐19 can be mapped to different columns on the PCM crossbar array. **Figure** **3**a shows the specific mapping method of CNN onto the PCM array, including input and output activations and weights. Since the conductance values are positive, while the weights may have both positive and negative values, each weight is typically mapped to the differential conductance of two adjacent 1T1R bit cells on different columns. The weights $W_{ij}^{k}$ for different convolutional kernels are represented by the corresponding differential conductance $G_{ij}^{k}$, expressed as follows.

(1) $$G_{ij}^{k}=G_{ij}^{k,+}\;-G_{ij}^{k,-}$$

where $G_{ij}^{k,+}$ and $G_{ij}^{k,-}$ are utilized to represent the conductance values of two PCM devices located in adjacent columns, indicating the weights. A linear mapping is essential between the weights of the CNN and the differential conductance of PCM devices, as specified in the following formula.

(2) $$G_{\mathrm{ij}}^{k}=G_{\max}\times \frac{W_{\mathrm{ij}}^{k}}{W_{\max}^{k}}+\delta G_{\mathrm{ij}}^{k}$$

Where *G_max_* is the programmable maximum conductance of the device, $W_{max}^{k}$ is the maximum weight value in the kernel, and $\delta G_{ij}^{k}$ denotes the error between the programmed conductance and the target conductance due to non‐ideal characteristics of the device.^[^ ^35^ ^]^ The non‐ideal characteristics mainly include noise during device programming and reading, variation between device and device, and conductance drift. When mapping the CNN weights, trained with FP32 precision, to simulate the analog conductance in PCM devices, $\delta G_{ij}^{k}$ is likely to lead to a significant decrease in the inference accuracy of the CNN due to the high precision requirements. Given that the noise in memristive devices and variation between device and device are considered to follow a Gaussian distribution, we address this issue by introducing additive Gaussian noise to the network weights during the network training. This is done to simulate the conductance error ($\delta G_{ij}^{k}$) introduced by noise in devices and variation between device and device during weight mapping, thereby enhancing the CNN inference accuracy. Previously, there have been a large number of approaches to injecting random Gaussian noise during training to improve the inference accuracy of CNN. However, it has been demonstrated that introducing an additive random Gaussian noise directly to the weights is a better and simpler way to simulate the behavior of PCM devices, eliminating the need for constructing overly complex noise models for precise hardware noise simulation. The addition of Gaussian random noise during the backward propagation process in network training does not effectively address this issue, and our PCM chip performs CNN inference, which is a forward operation. Hence, we only introduce additive Gaussian random noise to the weights during the forward process in the training phase to tackle the decrease in network accuracy caused by noise and variation in mapping CNN weights to PCM conductance. Since it is an additive Gaussian noise, the mean of the Gaussian distribution representing the noise is set to 0. Additionally, in order for this noise to better simulate the behavior of the PCM device, we determine the variance of this Gaussian distribution based on the behavior of the PCM device, as specified in the following formula.

(3) $$\frac{\sigma_{noise}^{k}}{W_{max}^{k}}\equiv \;\frac{\sigma_{G}}{G_{max}}=\alpha$$

Where $\sigma_{noise}^{k}$ represents the standard deviation of the Gaussian noise added within the specified kernel, σ_*G*_ denotes the standard deviation of the read and program noise measured in the PCM devices, and α is defined as a factor measuring the standard deviation of the noise. Here, we can adjust the Gaussian noise introduced to the weights based on the obtained α factor to achieve the optimal training effect. In this study, we experimented with various α factors and found that α = x worked the best for the DarkNet‐19 backbone. After training, we obtain a CNN that simulates the noise of the device, which can better mitigate the decrease in CNN inference accuracy caused by conductance errors. To better simulate the impact of device noise on CNN during hardware deployment, we also introduce additive Gaussian noise to the weights during inference (Figure 3c), with the standard deviation matching that of the additive Gaussian noise introduced during training.^[^ ^35^ ^]^

After training the DarkNet‐19 backbone to obtain the weights, we need to program the PCM device according to the mapped conductance values to achieve the purpose of deploying the CNN onto the PCM chip. The PCM device consists of a top electrode, a bottom electrode, and an intermediate phase‐change material, which is in the crystalline phase with a high conductance in its as‐fabricated state. Subsequently, a current pulse of sufficiently high amplitude, known as a RESET pulse, is applied to it, inducing melting in the majority of the intermediate phase‐change material due to Joule heating. After the pulse stops abruptly, the molten material undergoes quenching due to the glass transition, forming an amorphous region near the bottom electrode, and the device is in a low conductance state. Then a SET current is applied to the PCM device in its low conductance state, inducing crystallization in a portion of the amorphous region via Joule heating, leading to an increase in conductance.^[^ ^36^, ^37^ ^]^ Hence, the modulation of the current pulse applied to the PCM device allows for the control of its conductance, demonstrating the analog conductance characteristics inherent to the PCM device. Specifically, each individual PCM device has the capability to encode an analog range of values. In our study, leveraging the analog conductance characteristics of PCM devices enables the utilization of two differential PCM conductances to represent a single weight within the CNN.

The method for deploying CNN on a PCM chip involves a multi‐step process that begins with mapping the CNN model weights to the corresponding PCM device weights, which are then programmed onto the PCM chip. During computation, read pulses are applied to the PCM device, and the total current of each row is measured through the ADC, based on Kirchhoff's law. This current represents the sum of the currents through the various PCM cells, which corresponds to the outcome of the convolution operation—a key component of the matrix‐vector multiplication process in CNN. This vector is then passed through additional layers of the network for further processing, ultimately completing the entire CNN workflow. In this approach, we exploit the analog conductance characteristics of the PCM devices, effectively using them as both memory and computational units, allowing for efficient execution of VMM directly on the chip. This not only improves the overall speed of computation but also reduces the energy consumption typically associated with data transfer between separate memory and processing units.

In our proposed memristive in‐memory OD system, the specific system architecture of the in‐memory VMM module is illustrated in the figure, which has been validated as theoretically feasible. In the preceding sections, we have discussed how to mitigate the decrease in CNN inference accuracy caused by read and program noise of PCM devices, but still have not solved the degradation of CNN inference accuracy caused by PCM device conductance drift.^[^ ^38^ ^]^ Owing to the metastability of the amorphous state in the PCM device, there is a monotonic and time‐dependent decrease in conductance following programming, a phenomenon referred to as conductance drift.^[^ ^39^ ^]^ To address the conductance drift issue, we adopted the Global Drift Compensation method^[^ ^35^ ^]^ and designed a drift calibration unit (DCU), where the calibration process is as follows: 1) regularly perform inference using random samples from the training dataset; 2) select random columns from the PCM array, perform voltage readings, and compute the compensation factor based on the collected data; 3) apply the compensation factor globally across the system to correct for drift. The DCU thus compensates the conductance drift repeatedly at given time intervals. In the PCM chip, the array size of each core is designed as 1024 × 1024, and the memory array and capacity requirements of each layer in the backbone DarkNet‐19 network are shown in Figure S5 (Supporting Information). Therefore, we believe that our chip is capable of supporting the deployment of the DarkNet‐19 backbone. The PCM‐based in‐memory computing core architecture is shown in Figure 3b. The architecture of the PCM‐based in‐memory VMM module is shown in Figure 3b. It primarily consists of a 1T1R array, and the peripheral circuitry of the array includes word Lines (WL), source Lines (SL), bit Lines (BL) decoder, MUX, and driver circuits. The driver circuit and MUX are controlled by the decoder circuit, connected to the ports of the PCM array in order to perform operations such as reading and writing to PCM devices. During operation, analog voltage signals are fed into the PCM array through the digital‐to‐analog converter (DAC), BL, MUX & Driver as inputs to the CNN, and the conductance values of PCM serve as the weights for the CNN. Multiplication and accumulation operations are performed based on Ohm's Law and Kirchhoff's Law, resulting in an accumulated current, which is converted by the Trans‐Impedance Amplifier & ADC to the digital domain and is then input into the DCU, which can address conductance drift issues of PCM device induced by both time‐dependent and temperature‐dependent, ensuring stable weight values throughout inference. After undergoing drift calibration in the DCU, the data undergoes activation operations in the activation function unit, with the specific activation function determined by the CNN, using hardware‐friendly ReLU in the case of our proposed DarkNet‐19. The final output of the activation function unit is the output of the CNN inference.

Furthermore, we compare three parameter mapping (including weights and input/output) and their corresponding ADC configuration schemes for different blocks in the algorithm verification of the two CNN deployment methods (Figure 3d), and then develop a more efficient parameter mixed precision mapping scheme. The algorithm validation results of the three schemes of the first CNN deployment method are discussed in detail in the supplementary information. The results show that the performance of mixed‐precision mapping achieves similar accuracy as that of full high‐precision mapping, but with significantly reduced FLASH‐ADC and SAR‐ADC energy by 22.3 times and 2.9 times, respectively, with significantly better performance than full low‐precision mapping. In the in‐memory VMM module, all three schemes use W&V analog conductance values but employ different precision ADC (corresponding to the quantization precision of input/output) configuration schemes. Scheme A adopts 8‐bit ADC for all blocks; Scheme B adopts corresponding 8‐bit to 3‐bit ADC for Block1 ∼ 6; Scheme C adopts 3‐bit ADC for all blocks. We deploy the three schemes and evaluate the inference performance and energy efficiency via simulations based on experimental data on Cifar‐10. The measured drift coefficient v of each state is used in the simulations (Figure 3e,f). The results show that Scheme B achieves similar accuracy to Scheme A (Figure 3e), while reducing the energy consumption of the FLASH‐ADC and SAR‐ADC by 22.3× and 2.9×, respectively (Figure 3f), demonstrating significantly better performance than Scheme C. Therefore, Scheme B using mixed precision for different blocks is optimal.

In this module, we initially address the non‐ideal characteristics of PCM devices, including read and program noise, by introducing an additive Gaussian noise based on the behavior of PCM devices to weights during training. This training method results in a CNN better suited for deployment on PCM hardware, leading to more inference‐accurate outcomes when deployed on PCM chips. To address the issue of decreased CNN accuracy due to PCM device conductance drift, we design a DCU within the architecture of the in‐memory VMM module, providing stability for CNN deployment on PCM chips. Furthermore, to minimize the demand for high‐precision ADC while maintaining network performance, we develop an efficient mixed‐precision parameter mapping scheme, which reasonably selects a configuration scheme with ADC of varying precision. In general, in this section, we have successfully developed an accurate, stable, and efficient in‐memory VMM module for the memristive in‐memory OD system.

### 2.4 In‐Memory Max Computation Module

**Figure 4:**
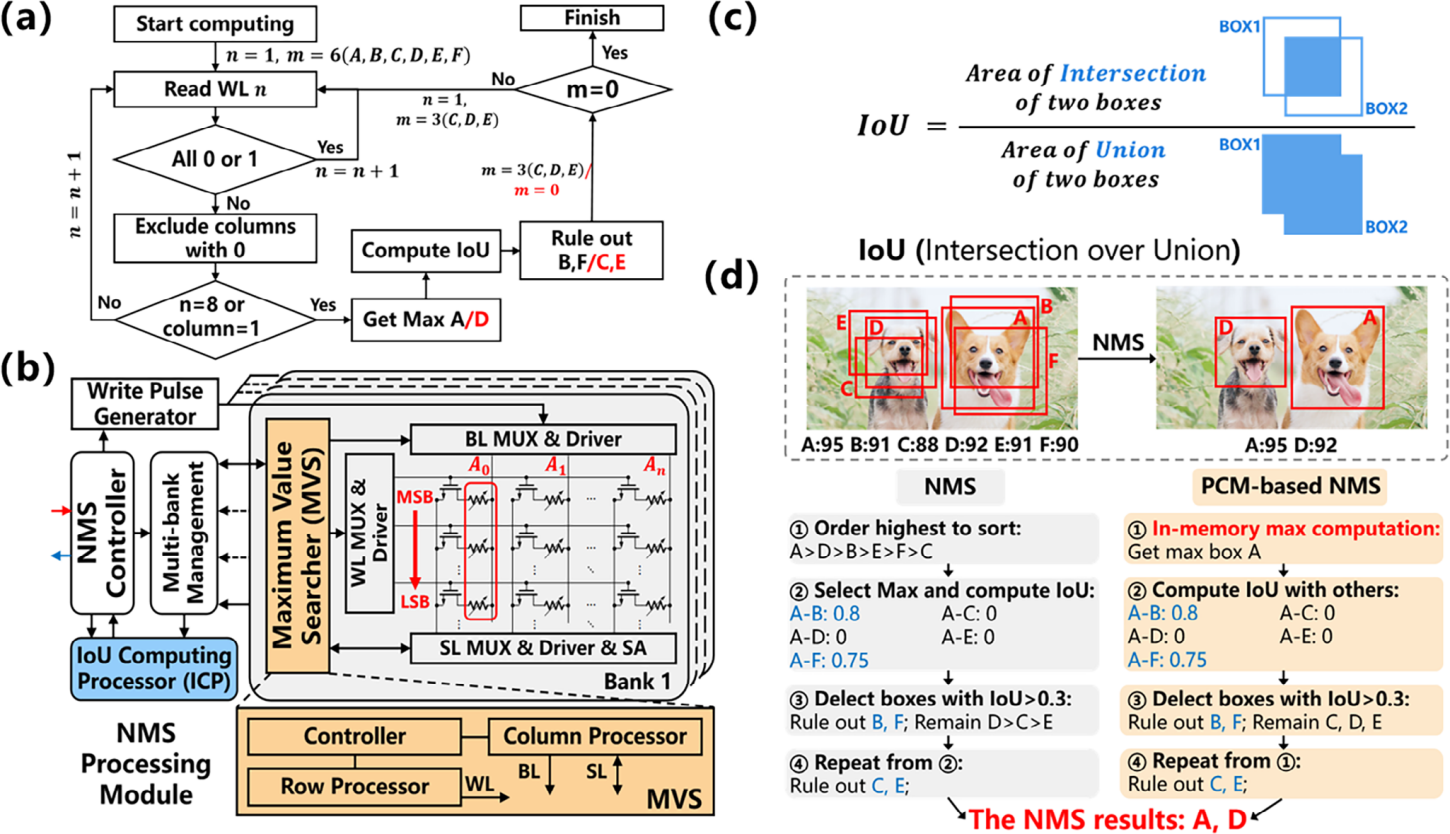
In‐memory max computation for PCM‐based OD. a) PCM‐based NMS flow chart, where the system stores data by column, and reads data by row. b) Illustration of PCM‐based NMS process. In‐memory max computation is used to identify the most confident bounding box, followed by iterative IoU‐based suppression of overlapping boxes. c) Visualization of (IoU computation, fundamental to NMS. IoU determines overlapping regions between bounding boxes (e.g., Box_1_ and Box_2_). d) Architecture of the NMS processing module. IoU computation and in‐memory max computation are efficiently performed within the ICP and memory banks.

After the prediction based on CNN, object localization is needed in the post‐processing step. This process primarily involves the use of the NMS algorithm, which eliminates redundant boxes from the candidate boxes determined by CNN and retains the optimal box. In the NMS algorithm, the main operational steps involve sorting the confidence scores of the candidate boxes predicted by CNN and searching for the maximum value. Subsequently, the remaining candidate boxes are iteratively compared with the box with the highest confidence score, calculating the IoU to remove duplicate boxes, i.e., boxes with IoU greater than a set threshold (**Figure** **4**c). In order to construct the PCM‐based memristive in‐memory OD system, we utilized the analog conductance characteristics of the PCM device to build an in‐memory VMM module, and in this section, we primarily leverage the binary conductance characteristics and high endurance of the PCM device to further design a PCM‐based in‐memory max computation module to accomplish the NMS processing module in memristive in‐memory OD system.

The architecture of our designed PCM‐based NMS processing module is shown in Figure 4b, consisting mainly of a PCM‐based in‐memory max computation module and peripheral functional circuits. The in‐memory max computation module mainly includes a Maximum Value Searcher (MVS), which contains a controller of Row Processor (WL) and Column Processor (BL & SL),^[^ ^40^ ^]^ the PCM array, the necessary MUX and driver circuits, as well as a Sense Amplifier. The peripheral circuits mainly include an NMS controller, a Write Pulse Generator, and an IoU Calculation Processor (ICP). The primary function of the NMS processing module is to store the confidence scores of boxes processed by CNN and execute the NMS operation to complete the OD task. While NMS performs the function, data (confidence scores of boxes) are input into the NMS controller, which then sends information to the Write Pulse Generator unit for the generation of write pulses, the generated write pulses are subsequently fed into the array through BL, programming the data to be ranked into the array. Subsequently, the MVS selectively activates devices on the array and issues a read pulse, executing an in‐memory max computation strategy to rank the data stored on the array and obtain the maximum value. The obtained maximum value is input into the peripheral functional circuit near the PCM array, specifically the ICP, to calculate the IoU. Finally, based on the calculated IoU, redundant boxes are removed, completing the remaining operations of the NMS algorithm.

In the PCM‐based NMS module, in‐memory max computation is achieved by the PCM array to realize MVS, which is a fundamentally different operation from VMM, and performing it in memory can significantly reduce data handling overheads. The difference between PCM‐based NMS and traditional NMS is that complex sorting operations are replaced by in‐memory max computations using PCM (Figure 4d). Assuming two objects yield three boxes each after undergoing CNN processing, the NMS operation is required to obtain the optimal detection boxes for the two objects. Confidence scores obtained from CNN processing are stored in the form of 8‐bit data in various columns of the PCM array, with each score represented by 8 PCM devices in the same column. The strategy of the PCM‐based NMS module is shown in Figure S6 (Supporting Information), utilizing in‐memory max computation to identify the two maximum numbers corresponding to the two objects from the six numbers, which are A: 95, B: 91, C: 88, D: 95, E: 91, F: 90, where A, B&F represent the detection boxes for object 1, and C, D&E represent the detection boxes for object 2. The first device in the PCM devices storing data in each column is denoted as the Most Significant Bit (MSB), and the last device is denoted as the Least Significant Bit (LSB). When the PCM‐based in‐memory max computation starts, the data in each row of the array are read by WL, and if the values of the row are all 0s or 1s, the row is skipped, and the next row is read (Steps #1‐#5). When a row is encountered with values other than all 0s or all 1s, the columns with values of 0 are excluded (Step #6). Repeat the above operation and when only one column is left or LSB is read, the remaining column is the maximum value of the current round of ranking (Step #7), following this method, a second round of ranking can be performed to find the second maximum value. The two maximum values obtained after two rounds of ranking (A & D) are then input into the ICP unit for further processing, and the boxes with IoU exceeding the threshold are removed to obtain the NMS results, i.e., the detection boxes (A & D) corresponding to each object (Step #8‐#15). The in‐memory max computation primarily involves programming PCM devices into binary states, and the endurance and latency of PCM devices will be discussed in the Supplementary Information. Figure 4a illustrates the flowchart of the PCM‐based NMS operation. When the calculation begins, data are read row by row from MSB to LSB, searching for the optimal detection box based on confidence scores. The controller then passes the maximum box name with the highest confidence score to the ICP and performs IoU calculations. Following this step, redundant boxes are deleted based on the IoU threshold, thereby finalizing the PCM‐based NMS operation.

### 2.5 The Application of Memristive In‐Memory OD System

OD algorithms have found numerous representative real‐world applications, and they can be used to address plenty of significant problems in the fields of scientific research and industrial production, i.e., utilizing classical OD algorithms to accomplish OD tasks. OD algorithms have been extensively utilized in industrial production and daily life for tasks such as object recognition, face detection, industrial detection, people counting, etc. Additionally, it has been discovered that OD algorithms hold significant applicability in the field of scientific research, particularly within the realm of neuroscience. This is exemplified by the development of Deepsqueak, which is based on OD algorithms and serves as a research tool that facilitates the study of the neural mechanisms underpinning the languages of rodent, mammalian, and primate species. The development of Deepsqueak was motivated by the considerable time‐consuming nature of studying the vocalization processes of these animals, as their vocalizations extend beyond the human auditory threshold of 20 kHz.^[^ ^41^, ^42^ ^]^

Despite the widespread application of OD algorithms across a myriad of fields, these algorithms often encounter significant challenges in terms of high energy consumption and prolonged execution times due to their substantial model sizes. To surmount these challenges, the employment of in‐memory computing methodologies, predicated on advanced non‐volatile memory technologies such as PCM, has demonstrated considerable potential. By diminishing the distance between processing and storage units, this approach facilitates an enhancement in chip computational power and efficiency, thereby efficaciously addressing the issues related to energy consumption and processing velocity. Furthermore, the advanced non‐volatile memory devices, characterized by their high storage density, cost‐effectiveness, and multiple resistive states, have emerged as a pivotal avenue for augmenting chip performance and efficiency, particularly in the post‐Moore's Law era. Within the ambit of this technological innovation, PCM exhibits superior performance in terms of integration density and storage capacity, fulfilling the high‐performance storage requisites of object detection algorithms. Concurrently, the multi‐resistive states and binary storage characteristics of phase‐change materials provide a foundational physical basis for the realization of complex computational operations, such as multiplication‐accumulation and sorting operations. These attributes render PCM an ideal candidate for the construction of a memristive in‐memory OD system, offering a promising direction for research and technological development in this field.

**Figure 5:**
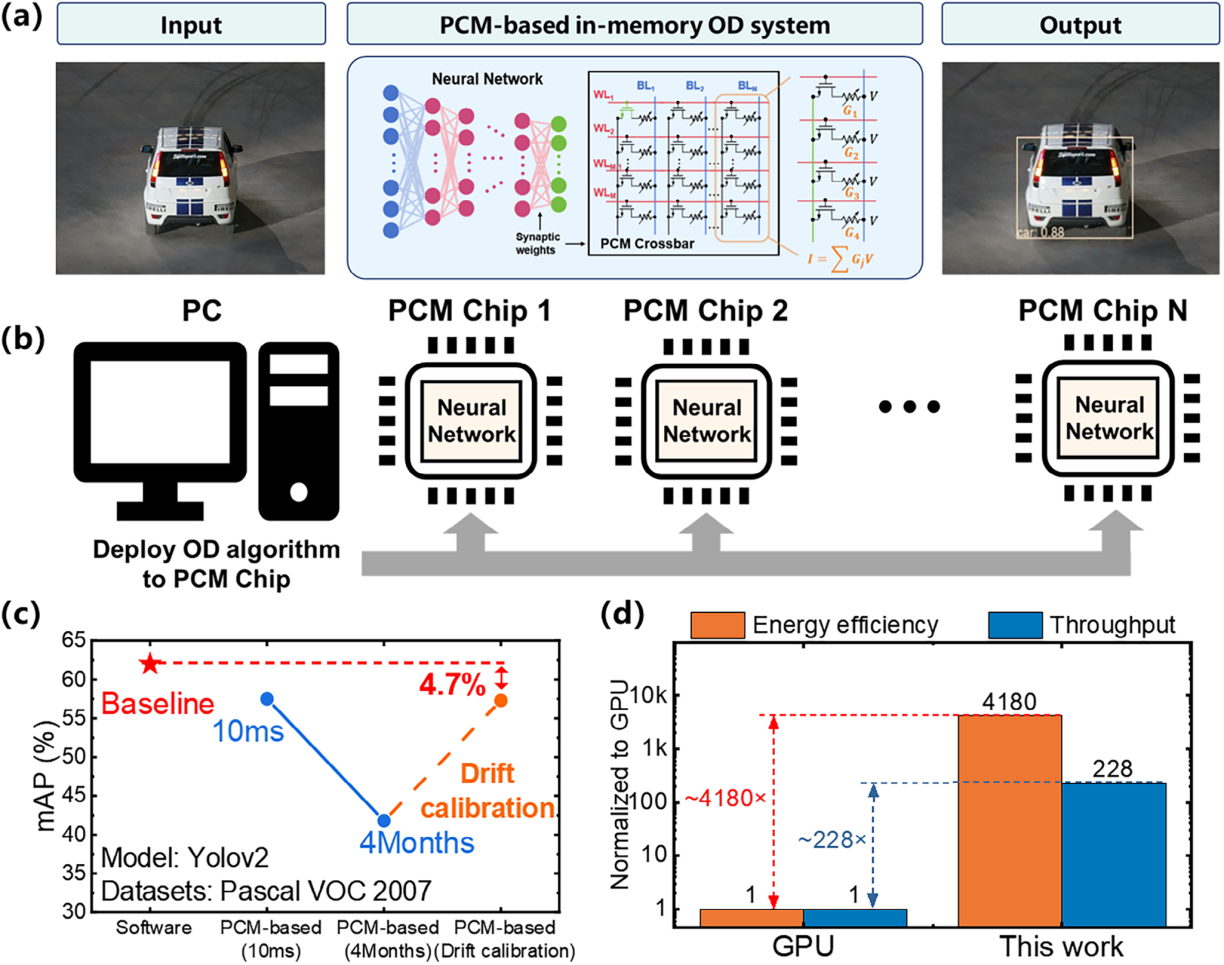
Performance and Benchmark of PCM‐based Object Detection System. a) The input image shows a car to be detected. The PCM‐based system architecture is illustrated, where a neural network processes the input data with synaptic weights stored in the PCM crossbar. The output image demonstrates the result of the detection, with a confidence score, such as “car 0.88,” displayed on the detected object. b) After training, the OD algorithm is deployed onto, potentially many, PCM chips, where the weights are located on the PCM crossbar. c) The graph shows the accuracy of the PCM‐based OD system over time, comparing the baseline and drift calibration. The accuracy is shown to improve with drift calibration after 4 months of deployment, achieving a 4.7% accuracy increase. d) The graph demonstrates that the PCM‐based OD system achieves a significant improvement in both time (228× faster) and energy 4180× more efficient) compared to baseline and drift calibration systems.

Currently, research on memristive in‐memory OD systems for OD using PCM remains unreported. Hence, we applied our memristive in‐memory OD system to the classical OD algorithms (YOLOv2) and performed a systematic demonstration of the classical object recognition task (**Figure** **5**a). Our results indicate that our memristive in‐memory OD system, when applied to the YOLOv2 algorithm (Figure 5b), achieves excellent performance, showcasing its strong potential applications in tools developed based on the YOLOv2 algorithm. Meanwhile, we have also conducted experimental demonstrations to validate the feasibility of applying the memristive in‐memory OD system in the classical OD algorithms (YOLOv2). Our findings demonstrate that the use of the memristive in‐memory OD system achieves results closely comparable to those of software‐based solutions, across both daily‐life and industrial object recognition tasks. The high compatibility of the memristive in‐memory OD system with compute‐in‐memory based on memristors crossbar array further highlights its enormous potential in the development of research tools that combine OD algorithms and CNN.

### 2.6 OD Performance and Benchmark

The demonstration above illustrates that the memristive in‐memory OD system is highly effective in executing tasks across industrial and scientific domains. This scenario paves the way for exciting new avenues in developing energy‐efficient OD systems. The memristive in‐memory OD system eliminates the most energy‐intensive aspect of traditional von Neumann architecture: the inherent extensive data transfer. We compared the performance of such PCM networks on OD tasks using the Pascal VOC 2007 dataset (Figure 5c). Our study reveals that while the accuracy of networks deployed on PCM arrays slightly decreases compared to their software‐based counterparts, with a modest reduction of only 4.5%, the presence of conductance drift in PCM devices still poses a significant challenge to network accuracy. It is observed that the impact of conductance drift on network accuracy intensifies over time, leading to a decline of more than 15% in network performance four months after programming the PCM devices. Such a loss in accuracy represents a substantial challenge, underscoring the need for strategies to mitigate the effects of conductance drift on the long‐term reliability of PCM‐based computing systems. While conductance drift can deteriorate network performance, the performance is significantly improved after DCU calibration, resulting in an accuracy that is only 4.7% lower than software and merely 0.2% lower than pre‐drift levels. This indicates that calibration via the DCU module can effectively mitigate the impact of conductance drift on network accuracy. In our final analysis, we conducted a detailed comparison between the energy efficiency of the memristive in‐memory OD system and traditional von Neumann architectures, primarily focusing on GPU‐based systems. Our findings indicate that our memristive in‐memory OD system, powered by PCM technology, significantly outperforms GPU‐based OD systems in terms of power consumption and operational speed. The energy efficiency and throughput of PCM‐based object detection systems are enhanced by 4180× and 228×, respectively, compared with NVIDIA A100 GPU^[^ ^43^ ^]^ (Figure 5d), while maintaining a small accuracy loss. This highlights the substantial advantages of in‐memory computing in enhancing the performance and energy efficiency of OD tasks.

## 3 Conclusion

OD algorithms are a type of artificial intelligence widely used in both industrial production and scientific research, with an architecture that is particularly well‐suited to the increasingly popular in‐memory computing technology. In this study, we introduce the concept of the memristive in‐memory OD system and present a 128Mb PCM chip for demonstration, which maintains a high memory yield rate of 99.99999%. For the first time, we have used a PCM chip for both in‐memory VMM and in‐memory max computation as two fundamental operations for OD operation, showcasing the capabilities of a PCM‐based memristive in‐memory OD system. In the proposed memristive in‐memory OD system, we have designed two integral modules for in‐memory VMM and in‐memory max computation. These modules are essential in mapping the core components of OD algorithms onto PCM chips, thereby operationalizing the memristive in‐memory OD system.

In this study, we present a 128Mb PCM chip with a high memory yield of 99.99999%. For the first time, we have used a PCM chip for both in‐memory VMM and in‐memory max computation as two fundamental operations for object detection. A novel mixed‐precision weight mapping is used, which reduces the energy consumption by 22.3× while maintaining the network performance. The energy efficiency and throughput of our PCM‐based OD system outperforms GPU by 4180× and 228×, respectively.

## 4 Experimental Section

### PCM Device

The PCM devices utilized in this work were fabricated in a 40 nm node. The PCM is fabricated between the TiN adhesion layer and the blade‐shaped bottom electrode contact. By adjusting the thickness of the deposited material, the width of the heating electrode can be precisely controlled, ensuring sufficient temperature generation for phase transitions in C‐GST during programming. The width of the heating electrode is 4 nm.

The width of the heating electrode significantly impacts the power consumption and yield of PCM. On one hand, we precisely control the growth thickness of the thin‐film material for the heating electrode using CVD, ensuring consistent electrode width in mass‐produced chips. On the other hand, a protective cladding layer is deposited on the surface of the heating electrode to prevent oxidation or corrosion during chip processing or application.

### Test Platform

PCM chips require parallel testing, necessitating automatic test equipment that supports multi‐channel operation, integrates both signal generation and measurement functions, and possesses data processing capabilities. In this study, the Magnum II test system, manufactured by Teradyne in the United States, was utilized. This system offers advanced arbitrary pattern generation and measurement capabilities, enabling the creation of customized pulse sequences for PCM chip programming. Subsequently, the resistance values of the PCM chips can be read and analyzed for data evaluation.

### PCM‐Based Yolov2 Algorithm Inference Simulator

A PCM‐based YOLOv2 algorithm inference simulator within a Python3 software simulation environment was developed to test the effectiveness of object detection using the YOLOv2 algorithm on PCM arrays. We opted for Google's PyTorch deep learning framework for simulator development, benefiting from its extensive library of algorithms, including native implementations of required activation functions and batch normalization. Moreover, any standard PyTorch code for DNNs can be seamlessly ported to our simulator, facilitating customized PyTorch operations. Our simulator incorporates non‐ideal hardware characteristics such as IR drop, programming noise, and read noise, implemented in PyTorch as previously described. It also allows for the input of PCM conductance data measured from hardware for inference with measured device data. Data converters at the crossbar input and output simulate digital quantization with adjustable precision, and a drift compensation module has been added post‐quantization at the output. Additionally, our simulator can quantize network weights with adjustable precision to emulate weight inference using the binary characteristics of PCM.

### Implementation of Proposed DarkNet‐19 Hardware‐Aware Training

The architecture of the DarkNet‐19 network, primarily follows the classical structure, featuring 19 convolutional layers and 5 max pooling layers. Specifically, it includes 12 convolutional layers with 3×3 kernels and 7 layers with 1×1 kernels, utilizing Leaky‐ReLU as the activation function. In this study, we slightly modified the original DarkNet‐19 implementation to facilitate hardware‐aware training. In order to simplify the hardware implementation of the activation function, we replaced Leaky‐ReLU with the more hardware‐friendly ReLU, yielding a modified DarkNet‐19 architecture. The input data is processed through six distinct DarkNet blocks into a 7×7×1024 feature map, which is then passed through a convolutional layer and average pooling layer, culminating in classification via softmax output.

Previous research has demonstrated that employing hardware‐aware training methods during software‐based network training can significantly enhance performance when networks are deployed for simulation computations on devices. Hardware‐aware training involves accounting for non‐ideal characteristics of hardware during the training process, specifically by introducing additive Gaussian noise, similar in magnitude and programming noise characteristics to the weights matrix during the forward pass of training. Additionally, we quantize input and output activations to better simulate the effects of ADC and DAC, including cycle‐to‐cycle read noise, output noise, and quantization effects present in hardware devices. While our study does not account for other factors such as ADC non‐linearity, they could be easily integrated. Subsequently, our weight programming framework can optimally translate these trained weights into analog hardware weights.

### Implementation of YOLOv2 Hardware‐Aware Training on Pascal VOC 2007

The widely utilized object detection algorithm YOLOv2 was selected for classification tasks on the Pascal VOC 2007 dataset. During the training of YOLOv2, its backbone was replaced with our enhanced version of the DarkNet‐19 network. Subsequently, aligning with the memristive in‐memory OD system concept, hardware‐aware training of the entire YOLOv2 network was conducted on the Pascal VOC 2007 dataset. Each training batch comprised 16 images, and multi‐scale training was initiated, varying the input size at predetermined steps and adjusting the scale of input images based on specified epoch values during different training phases, with both training and testing image dimensions set to 416 × 416. Following this, the same hardware‐aware training technique used on the modified DarkNet‐19 was applied to train YOLOv2, enhancing its adaptation to the various non‐ideal characteristics present in hardware.

### Mapping Synaptic Weights to Memory Arrays

After conducting hardware‐aware training on the network, the trained synaptic weights are programmed into the PCM array through a specific mapping approach that linearly correlates the range of weights to the conductance range of the devices. Each weight is represented by two devices: one for positive weights and the other for negative weights. This method ensures a precise and efficient translation of trained weights into the hardware, enabling the effective implementation of neural networks on PCM technology.

## Conflict of Interest

The authors declare no conflict of interest.

## Supporting information



Supporting Information

## Data Availability

The data that support the findings of this study are available from the corresponding author upon reasonable request.
