# Supplementary material for: Memristive In‐Memory Object Detection with 128 Mb C‐Doped Ge2Sb2Te5 PCM Chip
Source: Adv Sci (Weinh). 2025 Jul 17;12(36):e05678. doi: 10.1002/advs.202505678 (PMC12463051; doi:10.1002/advs.202505678)
Supplement: Supplementary file 1 — Supporting Information [file ADVS-12-e05678-s001.docx]

Supporting Information

**Memristive In-Memory Object Detection with 128 Mb C-doped Ge_2_Sb_2_Te_5_ PCM Chip**

Chenchen Xie^#^, Yuqi Li^#^, Longhao Yan^*^, Sannian Song, Houpeng Chen, Ruijuan Qi, Xi Li^*^, Yihang Zhu, Lianfeng Yu, Bonan Yan, Yaoyu Tao, Gaoming Feng, Yuchao Yang^*^, and Zhitang Song^*^

# Equally contributed authors.

**Supplementary section 1**

Object detection algorithms are primarily divided into two components: the feature extraction part, which is mainly based on matrix-vector multiplication (VMM) calculations, and the non-maximum Suppression (NMS) part, which primarily involves maximum value search operations^[1]^. In our proposed PCM-based in-memory object detection system, we fully leverage the binary and analog conductance characteristics of PCM to implement both VMM calculations and the maximum value search, thus building an efficient computational system. First, the feature extraction component, based on analog conductance of PCM device, is used to compute the scores of the bounding boxes. These scores are then stored in the PCM array using the binary characteristics of PCM. Finally, efficient NMS operations are performed through an in-memory search for the maximum value stored in the PCM, enabling optimized performance.


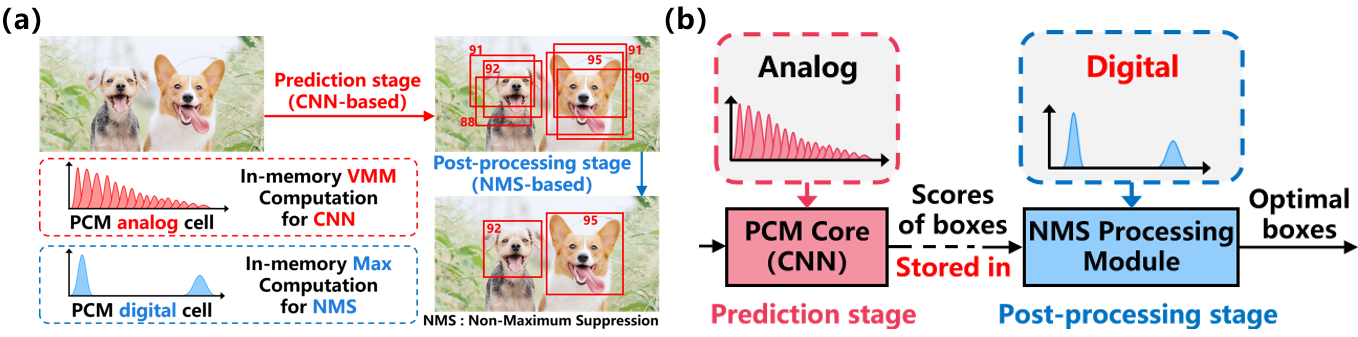


**Figure S1.** Introduction to phase change memory (PCM)-based object detection and applications. a) In this work, we combine PCM analog cells and digital cells to implement prediction and post-processing stage in object detection. b) The post-processing stage utilizes the NMS Processing Module to find optimal boxes.

**Supplementary section 2**

The Fig. S2 compares two different programming methods used in Phase Change Memory (PCM) systems: One-shot programming (OSP)^[2]^ and Write & Verify programming (W&V)^[3]^. In the OSP method, programming is performed in a single step, resulting in minimal delay but potentially significant overhead in terms of energy consumption, latency, and area. This method is typically faster, with delays on the order of microseconds, but the precision of the analog-to-digital converter (ADC) required can be much lower, leading to possible trade-offs in system performance. On the other hand, the W&V method involves a more complex process with verification steps, which introduces additional delay on the millisecond scale. However, this method provides higher ADC precision, which is beneficial for applications requiring more accuracy at the cost of increased programming time, energy consumption, and area usage^[4]^. The comparison highlights that while W&V offers higher precision, it comes with a 10^4^ to 10^5^ times longer programming time compared to OSP, leading to greater overheads in terms of energy, latency, and overall system complexity. This trade-off must be considered when designing PCM-based systems depending on the specific needs for speed, accuracy, and power efficiency.


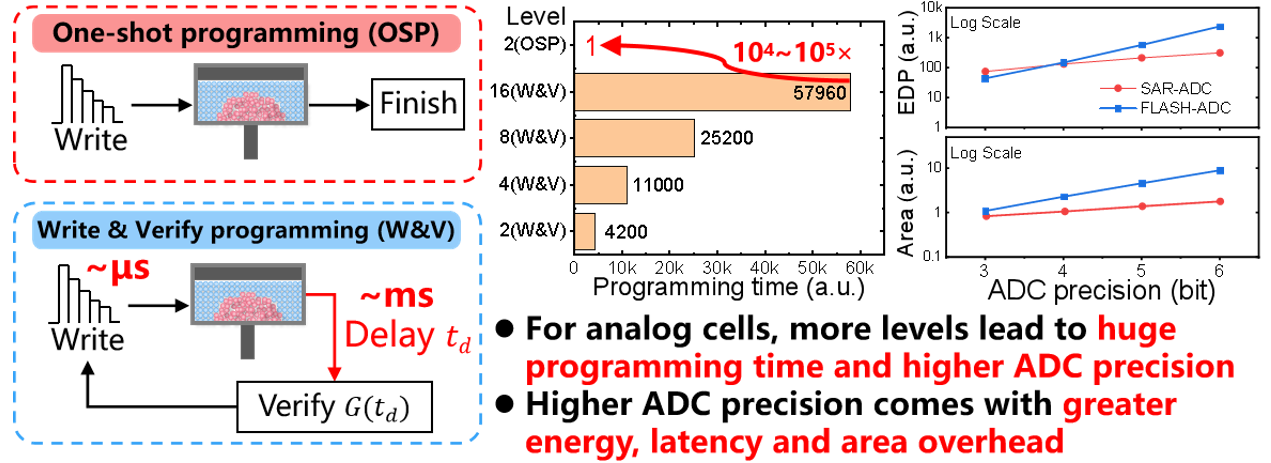


**Figure S2.** The relationship between state levels with programming time and ADC overheads.

**Supplementary section 3**

Fig. S3 presents a visual representation of the high yield and precision achieved in semiconductor manufacturing. On the left, a silicon wafer, showcasing the advanced production process. The right side of the figure represents the quality of individual chips on the wafer, with most of the chips showing a 100% yield rate, indicating flawless performance. However, one chip in the center has a slightly lower yield of 99.999475%. The overall yield is exceptionally impressive, reaching a staggering 99.99999%, demonstrating the reliability and efficiency of the manufacturing process. The manufactured phase-change memory chips are tested with the assistance of ATE and a probe. Every chip undergoes full-address write and read testing according to test patterns. Only devices whose read patterns match the test patterns are classified as good ones.


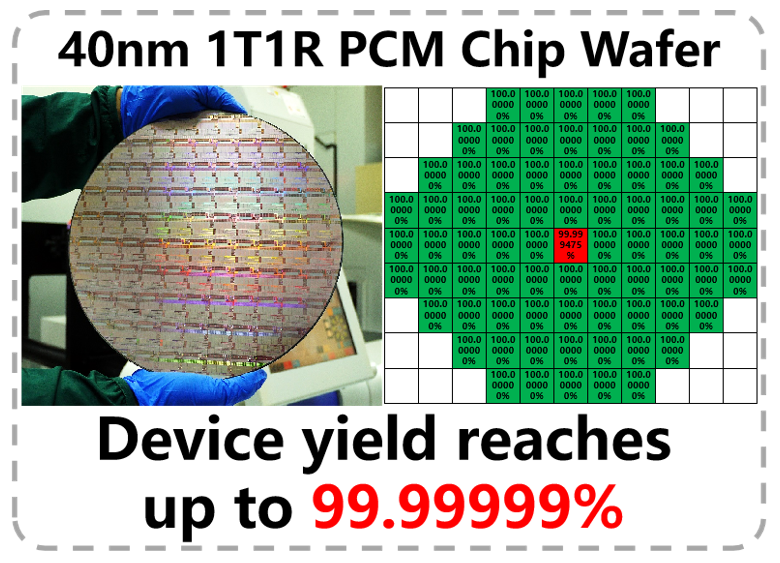


**Figure S3.** The picture of chip wafer and yield test results, with yield up to 99.99999%.

**Supplementary section 4**

The Fig. S4 demonstrates the conductance evolution of PCM devices under varying pulse durations (Tpulse) and current levels. Each curve represents a different Tpulse , illustrating how the conductance response varies with both pulse timing and current amplitude. As the SET current increases, the conductance exhibits multiple transition patterns, with longer pulse durations enabling higher conductance states. Notably, the diversity of conductance trajectories across different Tpulse values reveals the device's capability to achieve a wide range of conductance levels. This indicates that the PCM device possesses rich and finely tunable conductance states, which are essential for applications such as multi-level storage and analog in-memory computing[5].


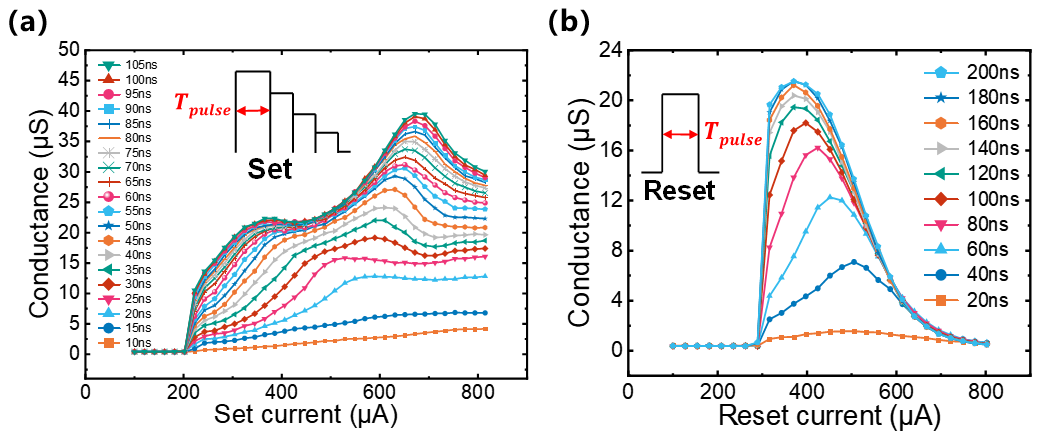


**Figure S4.** The relationship between state levels with programming time and ADC overheads. a) Conductance window diagram with different Set pulses. b) Conductance window diagram with different Reset pulses.

**Supplementary section 5**

Fig. S5 presents a strategy for mixed-precision weight mapping in neural networks^[6]^. The left side features a pie chart that displays the distribution of parameters across different layers. Layers 1–3 account for the largest proportion (68.1%), followed by layers 4–9 (25.1%), layers 10–14 (6.4%), and finally, layers 15–19 (0.3%). This distribution indicates that the early layers of the network contain the majority of the parameters, which is typical in many deep learning models.

On the right side, the mapping of weights to different ADC precision levels is outlined. Layers 1–3, which contain the most parameters, use 8-bit ADC precision to strike a balance between accuracy and computational efficiency. Layers 4–9 are assigned 6-bit precision, while layers 10–14 use 4-bit ADCs, and layers 15–19 are mapped to 3-bit precision. By employing lower precision for less critical layers, this mixed-precision approach optimizes both energy consumption and processing power.


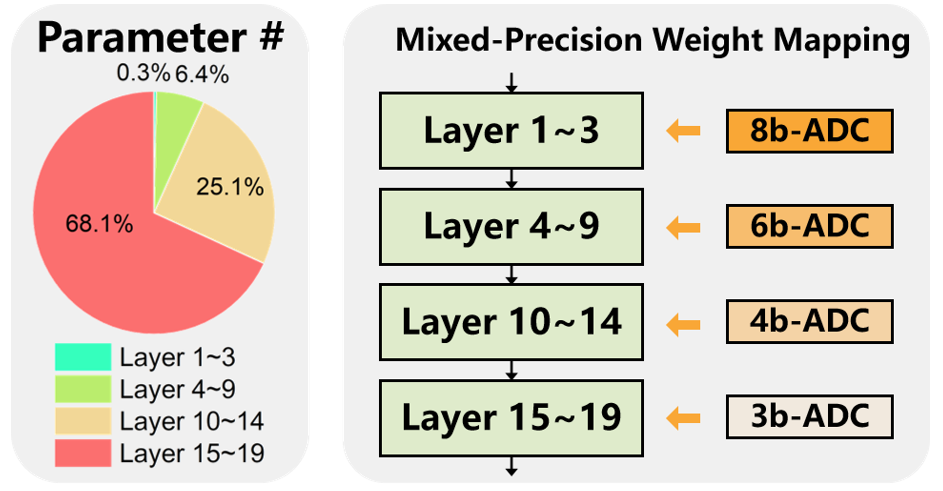


**Figure S5.** We fuse the two programming schemes and design mixed-level mapping and mixed-precision ADC configuration strategy, according to the parameter amount and precision sensitivity of different layers.

**Supplementary section 6**

Fig. S6 illustrates the process of non-maximum suppression (NMS) applied to bounding boxes in object detection. It begins by showing the scores for six bounding boxes (A, B, C, D, E, F), with their corresponding confidence scores displayed for each box. In the first step, box A has the highest score (95), so it is retained, and the boxes with significant overlap with A are eliminated, as shown by the 1 0 0 1 0 0 binary mask. This process effectively removes boxes B and F, which have substantial overlap with A. Next, the process proceeds to the next highest score, which is box D (92). After identifying the maximum score, box D is selected, and boxes C and E are ruled out due to their overlap with D, leaving A and D as the final selected boxes. The result of this NMS step is displayed as A & D, indicating that these are the bounding boxes retained after suppression. The key idea behind NMS is to reduce redundant detections by eliminating overlapping boxes and keeping only the most confident ones. This process is critical in object detection algorithms to ensure that the final output contains only the most relevant and non-redundant bounding boxes, improving the efficiency and accuracy of detection systems.


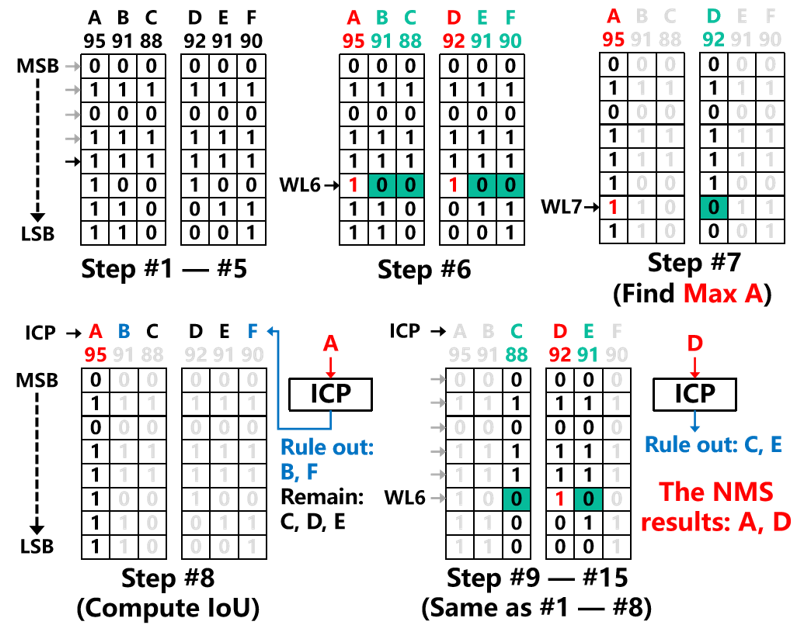


**Figure S6.** PCM-based NMS flow chart, where the system stores data by column, reads data by row.

**Supplementary section 7**

**PCM-based in-memory object detection system energy and time consumption**

The PCM-based in-memory object detection system includes two modules: in-memory VMM module and in-memory max computation module. We utilize 24 cores in in-memory VMM module, with each core comprising 2 1T1R PCM arrays, digital-to-analog converters (DAC), analog-to-digital converters (ADC), drivers, and digital activation units, etc. In-memory max computation module primarily consists of 1 array, sense amplifiers (SA), drivers, controllers, among other components.

The energy consumption and execution time of PCM-based in-memory system are calculated as follows:

$$W_{pcm-array}=G\times V^{2}\times T\times N=10\mu S\times{0.2V}^{2}\times28ns\times1024\times1024=11744pJ$$

$W_{pcm-array}$ is the energy consumption of a single PCM array. The size of the PCM array in core is 1024×1024, the average conductance of PCM is 10 μS, the average read voltage is 0.2 V, and the average time required for the array to complete the calculation is 28 ns^[4]^.

$$W_{core-DAC}=W_{DAC}\times N_{DAC}=0.015pJ\times1024=15.36pJ$$

$W_{core-DAC}$ is the energy consumption of all DACs corresponding to a single array, $W_{DAC}$ is the energy consumption of one DAC^[7]^, and $N_{DAC}$is the number of DACs corresponding to a single array (depending on BL).9

$$W_{B-ADC-8-SAR}=W_{SAR-ADC-8}\times N_{B-ADC-8}=207.2pJ$$

$$W_{B-ADC-6-SAR}=W_{SAR-ADC-6}\times N_{B-ADC-6}=3146pJ$$

$$W_{B-ADC-4-SAR}=W_{SAR-ADC-4}\times N_{B-ADC-4}=7402.8pJ$$

$$W_{B-ADC-3-SAR}=W_{SAR-ADC-}\times N_{B-ADC-3}=13408pJ$$

$W_{B-ADC-SAR}=W_{B-ADC-8-SAR}+ W_{B-ADC-6-SAR} +W_{B-ADC-4-SAR} + W_{B-ADC-3-SAR}$ = $24164pJ$

The total energy consumption of the SAR ADCs of B scheme for each precision level is calculated by multiplying the energy consumption of each individual SAR ADC with the corresponding number of ADCs for that precision^[4]^. For instance, the total energy consumption for the 8-bit SAR ADCs,

$W_{B-ADC-8-SAR}$, is obtained by multiplying the energy per 8-bit SAR ADC, $W_{SAR-ADC-8}$, with the number of 8-bit SAR ADCs, $N_{B-ADC-8}$, resulting in 207.2 pJ. Similarly, for the 6-bit SAR ADCs, the total energy $W_{B-ADC-6-SAR}$is 3146 pJ, while for the 4-bit and 3-bit SAR ADCs, the total energies are 7402.8 pJ and 13408 pJ, respectively. The overall energy consumption for all the SAR ADCs in the B scheme, $W_{B-ADC-SAR}$, is the sum of these individual contributions, yielding a total of 24164 pJ. This calculation highlights the cumulative power requirements for the mixed-precision ADC configuration of B scheme, with lower-bit ADCs contributing significantly to the total energy consumption.

$$W_{C-ADC-SAR}=W_{SAR-ADC-3}\times N_{ADC}=19660.8pJ$$

$$W_{A-ADC-SAR}=W_{SAR-ADC-8}\times N_{ADC}=1.4pJ\times49152=68812.8pJ \boldsymbol{(}\boldsymbol{2.9}\boldsymbol{\times}\boldsymbol{W}_{\boldsymbol{B-ADC-}\boldsymbol{SAR}}\boldsymbol{)}$$

The total energy consumption for the SAR ADCs in the C scheme, $W_{C-ADC-SAR}$, is calculated by multiplying the energy per 3-bit SAR ADC, $W_{SAR-ADC-3}$, by the number of ADCs, $N_{ADC}$ . This results in a total energy consumption of 19660.8 pJ. In comparison, the total energy consumption for the SAR ADCs of A scheme, $W_{A-ADC-SAR}$, is obtained by multiplying the energy per 8-bit SAR ADC, $W_{SAR-ADC-8}$, by the same number of ADCs, yielding a total of 68812.8 pJ, which is 2.9 times greater than the energy consumption of the SAR ADCs of C scheme. This calculation underscores the significantly higher energy demands of the A scheme due to its use of higher precision ADCs, highlighting the trade-offs between precision and energy consumption in different ADC configurations.

$$W_{B-ADC-8-FLASH}=W_{FLASH-ADC-8}\times N_{B-ADC-8}=9472pJ$$

$$W_{B-ADC-6-FLASH}=W_{FLASH-ADC-6}\times N_{B-ADC-6}=44044pJ$$

$$W_{B-ADC-4-FLASH}=W_{FLASH-ADC-4}\times N_{B-ADC-4}=37014pJ$$

$$W_{B-ADC-3-FLASH}=W_{FLASH-ADC-3}\times N_{B-ADC-3}=50280pJ$$

$W_{B-ADC-FLASH}=W_{B-ADC-8-FLASH}+ W_{B-ADC-6-FLASH} +W_{B-ADC-4-FLASH} + W_{B-ADC-3-FLASH}=140810pJ$

The total energy consumption of the FLASH ADCs of B scheme for each precision level is calculated by multiplying the energy consumption of each individual FLASH ADC with the corresponding number of ADCs for that precision. For instance, the total energy consumption for the 8-bit FLASH ADCs,

$W_{B-ADC-8-FLASH}$, is obtained by multiplying the energy per 8-bit FLASH ADC, $W_{FLASH-ADC-8}$, with the number of 8-bit FLASH ADCs, $N_{B-ADC-8}$, resulting in 9472 pJ. Similarly, for the 6-bit FLASH ADCs, the total energy $W_{B-ADC-6-FLASH}$is 44044 pJ, while for the 4-bit and 3-bit FLASH ADCs, the total energies are 37014 pJ and 50280 pJ, respectively. The overall energy consumption for all the FLASH ADCs in the B scheme, $W_{B-ADC-FLASH}$, is the sum of these individual contributions, yielding a total of 140810 pJ.

$$W_{A-ADC-FLASH}=W_{FLASH-ADC-8}\times N_{ADC}=3145728pJ$$

$$W_{C-ADC-FLASH}=W_{FLASH-ADC-3}\times N_{ADC}=73728pJ$$

The total energy consumption for the FLASH ADCs in the C scheme, $W_{C-ADC-FLASH}$, is calculated by multiplying the energy per 3-bit FLASH ADC, $W_{FLASH-ADC-3}$, by the number of ADCs, $N_{ADC}$. This results in a total energy consumption of 73728 pJ. In comparison, the total energy consumption for the FLASH ADCs of A scheme, $W_{A-ADC-FLASH}$, is obtained by multiplying the energy per 8-bit FLASH ADC, $W_{FLASH-ADC-8}$, by the same number of ADCs, yielding a total of 3145728 pJ, which is 22.3 times greater than the energy consumption of the FLASH ADCs of C scheme.

$$W_{core-driver}=W_{driver}\times N_{driver}=0.01pJ\times1024\times3=30.72pJ$$

$W_{core-driver}$ represents the total energy consumption of all drivers associated with a single PCM array, $W_{driver}$ is the power consumption of an individual driver, and $N_{driver}$ is the number of drivers corresponding to a single array, which depends on the number of word lines (WL), bit lines (BL), and select lines (SL).

$$W_{core-digital}=200pJ$$

$W_{core-digital}$ represents the estimated power consumption of all digital circuits, which primarily include clock circuits, control circuits, digital activation, and the TEU. These circuits operate at a clock frequency of 200 MHz.

$$W_{core}={(W}_{pcm-array}{+W}_{core-DAC}+W_{core-driver})\times2+W_{core-digital}=24363.8pJ$$

$W_{core}$ represents the total energy consumption of the core, excluding the ADC. The core consists of 2 arrays and their peripheral circuits, with the digital circuits shared across the 2 arrays.

$$T_{core-delay}=T_{DAC}+T_{ADC}+T_{core-digital}=10ns+64ns+20ns=94ns$$

$T_{core-delay}$ represents the time consumption of the core, primarily including the time delays associated with the DAC, ADC, and digital circuits.

The energy consumption and time consumption of the NMS processing module are calculated as follows:

$$W_{NMS-array}=G\times V^{2}\times T\times N=10\mu S\times{0.2V}^{2}\times8ns\times1024\times1=3.28pJ$$

$W_{NMS-array}$ represents the energy consumption of the array. The array in the RMU has a size of 1024×1024, with an average conductance of the PCM set at 10 μS, and an average read voltage of 0.2 V. The time required for the array to complete the calculation is 5 ns. Since the PCM in the NMS processing module only utilizes binary characteristics of PCM device, there is no need for a high-precision ADC, resulting in lower time consumption.

$$W_{NMS-SA}=W_{SA}\times N_{SA}=0.3pJ\times1024=307.2pJ$$

$W_{RMU-SA}$ is the energy consumption of all SAs corresponding to the array, $W_{SA}$ is the energy consumption of one SA, and $N_{SA}$ is the number of SAs corresponding to the array.

$$W_{RMU-driver}=W_{driver}\times N_{driver}=0.01pJ\times1024\times3=30.72pJ$$

$W_{RMU-driver}$ is the energy consumption of all drivers corresponding to the array, $W_{driver}$ is the power consumption of one driver, and $N_{driver}$ is the number of drivers corresponding to the array (depending on WL, BL and SL)

$$W_{NMS-digital}=710pJ$$

$W_{RMU-digital}$ is the estimated power consumption of all digital circuits. Digital circuits mainly include clock circuits, controllers, the mutation unit and row & column processors, etc. These circuits work at a clock frequency of 200MHz.

$$W_{NMS}=W_{NMS-array}+W_{NMS-SA}+W_{NMS-driver}+W_{NMS-digital}=1051.2pJ$$

$W_{RMU}$ is the total energy consumption of RMU, mainly including the array, SAs, drivers and digital circuits.

$$T_{NMS-delay}=T_{SA}+T_{NMS-digital}=8ns+10ns$$

T is the total time consumption of RMU, mainly including the consumption time of SAs and digital circuits.

$$W_{pcm-total}=W_{B-ADC-SAR}+W_{core}\times24+W_{NMS}=6.1\times{10}^{-7}J$$

$$T_{pcm-total}=T_{core}+T_{NMS}=112ns$$

$W_{pcm-total}$ is the total energy consumption of the system (24 cores parallel operations), and $T_{pcm-total}$ is the time consumption of the system.

**GPU energy and time consumption**

$$T_{compute}=\frac{OPS}{Throughput}=\frac{1024\times1024\times2\times2\times24+1024\times1024\div32}{312\times{10}^{12}}=323ns$$

$T_{compute}$ is the time consumed by the GPU for computing NAS, using the total operations $OPS$ and Float32 computing power $Throughput$ for calculation^[8]^.

$$W_{compute}=P\times T_{compute}=100W\times323ns=3.23\times{10}^{-5}J$$

$W_{compute}$ is the energy consumption of GPU computing. Since the model is small, in order to evaluate energy consumption more reasonably, we take 25% of thermal design power (TDP) as the average power consumption of GPU^[9]^.

$$T_{movement}=\frac{Data}{Bandwidth}=\frac{1024\times1024\times2\times24+1024\times1024}{2039\times{10}^{9}}=25199ns$$

$T_{movement}$ is the time consumption of GPU data movement. Since GPU is a volatile processor, data needs to be reloaded every time.

$$W_{movement}=P\times T_{movement}=100W\times25199ns=2.52\times{10}^{-3}J$$

$W_{movement}$ is the energy consumption of GPU data movement, it can be seen that data movement takes up a lot of time and energy consumption.

$$W_{GPU}=W_{compute}+W_{movement}=2.55\times{10}^{-3}J \boldsymbol{(4}\boldsymbol{180}\boldsymbol{\times}\boldsymbol{W}_{\boldsymbol{pcm-total}}\boldsymbol{)}$$

$$T_{GPU}=T_{compute}+T_{movement}=25522ns \boldsymbol{(}\boldsymbol{228}\boldsymbol{\times}\boldsymbol{T}_{\boldsymbol{pcm-total}}\boldsymbol{)}$$

$W_{GPU}$ and $T_{GPU}$ are the energy consumption and time of implementing nas based on GPU respectively

**Supplementary section 8**

Figure S7 shows the temperature dependence of PCM cell resistance. As temperature rises from –40 °C to 85 °C, both the high-resistance state (HRS, black) and the low-resistance state (LRS, red) exhibit a clear negative temperature coefficient. The HRS resistance falls from roughly 10 MΩ at –40 °C to about 2 MΩ at 85 °C, while the LRS drops from approximately 200 kΩ to 50 kΩ over the same range (Fig. S7). This monotonic decrease highlights the importance of drift calibration mechanisms when using PCM-based in-memory computing. Building on this capability, our drift calibration unit (DCU) collects column-wise current measurements from the PCM arrays, computing correction factors for drift calibration, and applying them to counteract temperature-induced resistance drift. As a result, our study fully incorporates temperature-dependent variations in PCM resistance and demonstrates that the DCU offers a promising solution for suppressing temperature-driven drift.


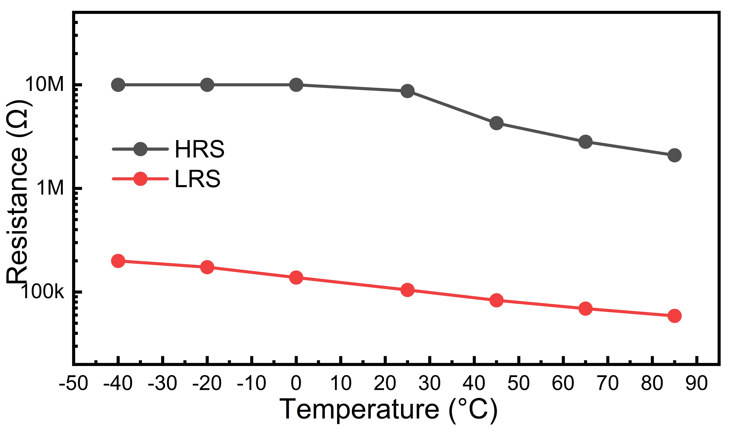


Figure **S7.** Resistance drift of PCM device in its high-resistance state (HRS, gray) and low-resistance state (LRS, red) as a function of temperature.

**Supplementary section 9**

Table S1 (shown below) lines up our 128 Mb PCM Chip in 40 nm node against other prior in-memory computation works. Our PCM chip excels in several practical aspects: it is fabricated in a 40 nm process, delivers a 128 Mb capacity, natively supports both VMM and in-memory maximum computation modes in hardware, and implements compensation for non-idealities, which is rarely offered together in existing in-memory computation works^[10-13]^.

Table S1 Comparison with prior in-memory computation works

**
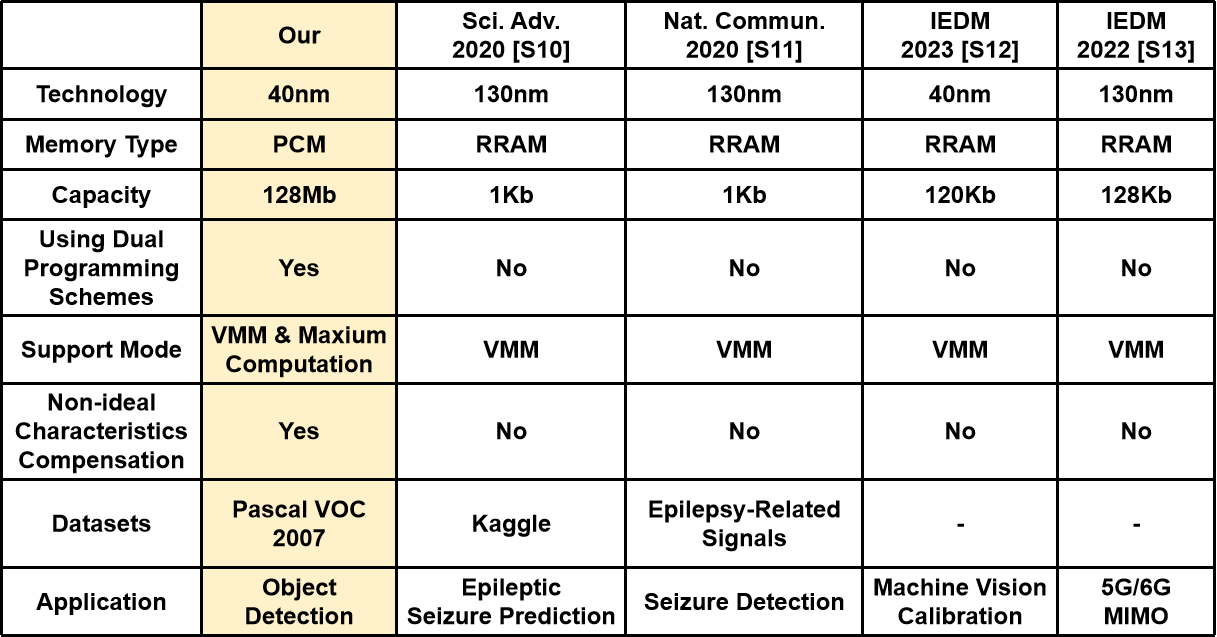
**

**Supplementary section 10**

In our memristive in-memory object detection architecture, some non-idealities introduce errors during computation: the noise of the large number of DACs/ADCs, and the conductance drift of the PCM cells. To quantify how each of these factors degrades computational accuracy, we analyze the resulting error in repeated vector-matrix multiplications (the primitive of the PCM-based in-memory computing). Based on our computational error analysis, our PCM chip is fully capable of supporting the construction of our memristive in-memory object detection (Fig. S8)

**
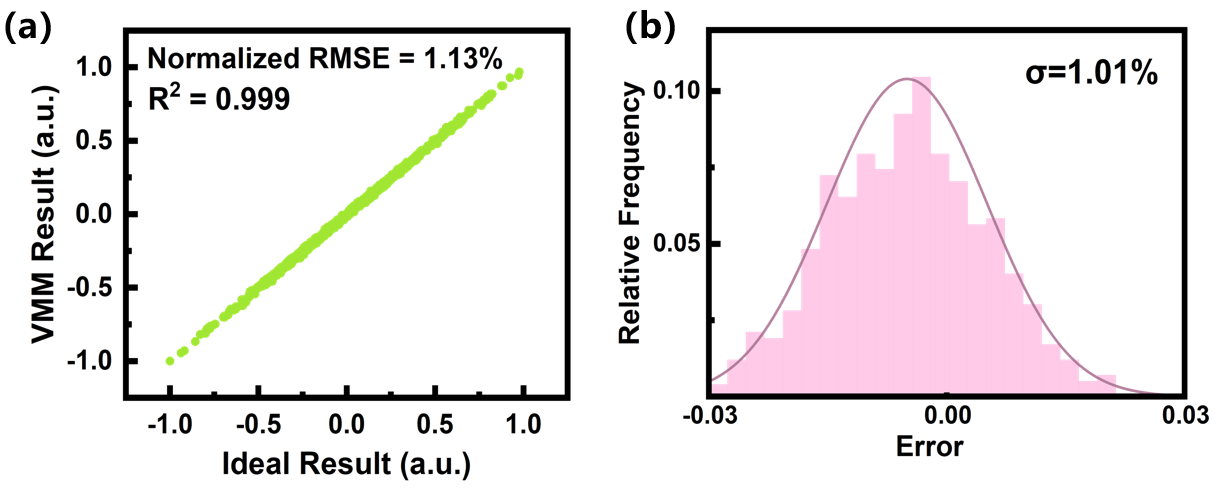
**

Figure **S8**. VMM Computation Accuracy Evaluation a) Comparison of normalized VMM outputs against the ideal dot-product results. b) Distribution of the VMM computation errors.

**Supplementary section 11**

The whole workload in object detection is not just vector matrix multiplication and max computation. Other operators in object detection, including ReLU activation, batch normalization, max-pooling, global average-pooling, Softmax, and IoU computation, can be fully realized using standard CMOS logic circuits.

Activation Function Layer. We replace the original Leaky-ReLU with the simpler, more hardware-friendly ReLU. In our memristive in-memory object detection architecture, the output currents of the PCM array are first converted to voltages by a transimpedance amplifier (TIA). A clamping element, either a diode or a gate-controlled MOSFET, is then connected in parallel at the TIA output. Whenever the voltage tends toward negative values, the clamp conducts, fixing the output at 0 V and thus implementing the ReLU in a single stage. This approach both simplifies the circuit and reduces area and power costs, providing an efficient on-chip ReLU solution.

Batch Normalization (BN) Layer. During training, BN learns scale and shift parameters for each channel, but these parameters remain constant during inference. Hence, we fuse the BN transform into the preceding layer by folding the learned scale and shift into the weights of the layer. The fused weights map directly onto PCM conductance, eliminating any need for dedicated BN hardware or additional control logic.

2×2 Max-Pooling Layer. To implement 2×2 max pooling, output feature-map data are streamed through a row buffer and sliding-window column registers that form each 2×2 patch in real time. Three comparators then perform two successive pairwise comparisons to select the maximum of the four inputs. With this scheme and pipelined buffering, each 2×2 max-pooling operation completes in just two clock cycles.

Global Average Pooling Layer. After convolution, feature map pixels are written, row by row, into a dual row buffer. Corresponding elements from the current and previous rows feed a three-stage adder tree that computes local sums each clock cycle; these partial sums accumulate in a per-channel accumulator register. Once all pixels in the map have been processed, the accumulator holds the total sum. A barrel shifter then right shifts this sum, and a lookup table applies a fine correction to yield the exact channel mean.

Softmax Layer. To normalize a vector into probabilities, we first find its maximum element via a comparator and subtract it in parallel from all inputs (preventing exponent overflow). An exponent lookup table then computes each element’s exponential. These values feed an adder tree to obtain their sum, and a reciprocal lookup table produces the reciprocal of that sum. Finally, a parallel multiplier array multiplies each exponential by the reciprocal to produce the Softmax output.

IoU Computation. Intersection-over-Union is implemented by comparators to identify overlapping bounding-box coordinates, subtractors to compute intersection dimensions, and adders to accumulate areas, followed by a hardware divider to compute the ratio of intersection to union.

**Reference**

[1] W. Tang, J. Chen, Y. Ning, K. Xu, presented at *2023 3rd International Conference on Electronic Information Engineering and Computer Science (EIECS)*, Changchun, China, September, **2023**.

[2] C. Xie, X. Li, H. Chen, Y. Li, Y. Liu, Q. Wang, K. Ren, Z. Song, *Micromachines* **2019**, 10.

[3] S. R. Nandakumar, I. Boybat, J.-P. Han, S. Ambrogio, P. Adusumilli, R. L. Bruce, M. BrightSky, M. Rasch, M. Le Gallo, A. Sebastian, presented at *2020 IEEE International Electron Devices Meeting (IEDM)*, San Francisco, USA, December, **2020**.

[4] H. Jiang, W. Li, S. Huang, S. Cosemans, F. Catthoor, S. Yu, *IEEE Design & Test* **2022**, 39, 48.

[5] L. Yan, Q. Wu, X. Li, C. Xie, X. Zhou, Y. Li, D. Shi, L. Yu, T. Zhang, Y. Tao, B. Yan, M. Zhong, Z. Song, Y. Yang, R. Huang, *Advanced Functional Materials* **2023**, 34.

[6] R. Joseph, F. Ali, presented at *Proceedings of the IEEE conference on computer vision and pattern recognition(CVPR)*, Honolulu, USA, July, **2017**.

[7] B. V. S. Cosemans, J. Doevenspeck, I. A. Papistas, F. Catthoor, P. Debacker, A. Mallik, D. Verkest, presented at *IEEE Int. Electron Devices Meeting (IEDM)*, San Francisco, USA, December, **2019**.

[8] NVIDIA A100 GPU data, <https://www.nvidia.com/en-us/data-center/a100/>, accessed: December, 2022.

[9] Y. Lu, X. Li, B. Yan, L. Yan, T. Zhang, Z. Song, R. Huang, Y. Yang, *Advanced Materials* **2021**, 34.

[10] J. T. Z. Liu, B. Gao, P. Yao, X. Li, D. Liu, Y. Zhou, H. Qian, B. Hong, H. Wu, *Nat. Commun.* **2020**, 11, 4234.

[11] J. T. Z. Liu, B. Gao, X. Li, P. Yao, Y. Lin, D. Liu, B. Hong, H. Qian, H. Wu, *Sci. Adv.* **2020**, 6, eabc4797.

[12] B. G. Q. Qin, Q. Liu, Z. Liu, Y. Lin, P. Yao, Y. Zhou, R. Yu, Z. Hao, J. Tang, Q. Zhang, L. Dai, Z. Su, Q. Xu, S. You, H. Wu, H. Qian, presented at *IEEE Int. Electron Devices Meeting (IEDM)*, San Francisco, USA, December, **2022**.

[13] Z. W. L. Bao, Q. Wang, Y. Yang, Y. Gao, L. Shan, J. Sun, Y. Yang, Y. Ling, H. Zhang, C. Wang, H. Xiao, L. Ye, A. Guo, L. Shen, W. Gu, G. Feng, C. Li, S. Chen, Y. Zhao, S. Huang, Y. Cai, R. Huang, presented at *IEEE Int. Electron Devices Meeting (IEDM)*, San Francisco, USA, December, **2023**.
